# Terrorist attacks: cutaneous patterns of gunshot and secondary blast injuries

**DOI:** 10.1080/20961790.2020.1771859

**Published:** 2020-07-01

**Authors:** Yann Delannoy, Isabelle Plu, Isabelle Sec, Tania Delabarde, Marc Taccoen, Antoine Tracqui, Bertrand Ludes

**Affiliations:** a Institut Médico-Légal de Paris, Paris, France; b CHU de LILLE, Lille, France; c Sorbonne Université, Paris, France; d Unité Médico Judiciaire, Hôtel Dieu, APHP, Paris, France; e Service de médecine légale, CHRU Besançon—Hôpital Saint-Jacques, Besançon, France; f Paris University, CNRS FRE 2029, Paris, France

**Keywords:** Forensic sciences, forensic medicine, blast injuries, explosive agents, suicide, gunshot wounds

## Abstract

Terrorist attacks have been on the rise. During the recent terrorist attacks in France, terrorists perpetrated their acts using weapons of war, as well as explosive charges. These two modes of action, when combined, can create skin lesions with similar macroscopic appearances, which can sometimes go unnoticed because of body fragmentation. A total of 68 autopsies, 83 external examinations, 140 standard radiographic examinations, and 49 computed tomography (CT) scans were performed over 7 days during the 2015 terrorist attacks in France. Bodies were injured by firearms and shrapnel-like projectiles. We analysed the clinical findings for the secondary blast cutaneous lesions from the explosive devices and compared these lesions with ballistic-related lesions to highlight that patterns can be macroscopically similar on external examination. Secondary blast injuries are characterised by penetrating trauma associated with materials added to explosive systems that are propelled by explosive air movement. These injuries are caused most often by small, shrapnel-like metallic objects, such as nails and bolts. Propulsion causes ballistic-type injuries that must be recognised and distinguished from those caused by firearm projectiles. Differentiating between these lesions is very difficult when using conventional criteria (size, shape, number and distribution on the body) with only external examination of corpses. This is why the particularities of these lesions must be further illustrated and then confirmed by complete autopsies and radiological and anatomopathological examinations.

**Key points**
- When occurring simultaneously in terrorist attacks, injuries caused by secondary blasts appear as cutaneous wound patterns that can be macroscopically very similar to those caused by firearm projectiles.
- The criteria usually found in the literature for distinguishing these two types of projectiles may be difficult to use.
- It is important in these difficult situations to benefit from systematic postmortem imaging.
- Systematic autopsy and then anatomopathological analyses of the orifices also help determine the cause of the wounds.

## Introduction

The number of terrorist acts involving bombs has multiplied in recent years (Madrid in 2004, London in 2005, Boston in 2013, Paris in 2015 and Brussels in 2016). In France, the global terrorism database recorded 241 deaths caused by terrorist attacks from 1970 to 2015. Since 2015, nearly 300 people have been victims of terrorism in France, as many as in the previous 40 years [1].

Forensic examinations in the context of acts of terror are increasingly frequent; however, lesions caused by explosive phenomena are rarely observed in routine practice. Therefore, forensic pathologists must be trained to recognise these specific wound patterns and their diagnoses [2]. In addition to the issue of victim identification [3, 4], it is fundamental to distinguish between the current modes of terrorist action.

Secondary blast injuries are not very specific because they result from projectiles, which can be varied and numerous. During recent attacks in France, the modus operandi of these acts of terror was the use of high-velocity firearms, sometimes with modified projectiles, together with explosives (charges or belts). The patterns of injury from firearms and projectiles expelled by blast waves are often very similar. It is these particular lesions and their diagnoses that we endeavour to present in our descriptions of clinical cases from recent experiences.

## Materials and methods

For this research, we collected data on terrorist attacks in France during 2015. Data were collected by our team within the French Forensic Institute of Paris.

A total of 68 autopsies, 83 external examinations, 140 standard radiographic examinations and 49 computed tomography (CT) scans were performed over 7 days in collaboration with the French Disaster Victim Identification team, following the International Criminal Police Organization’s (INTERPOL’s) protocols.

Bodies were injured by firearms and shrapnel-like projectiles. We collected the following data from 151 reports of entrance wounds: injury type, size, shape, number and distribution. Each injury was photographed, and we collected anatomopathological samples and residue samples.

## Results

**Figure 1.**
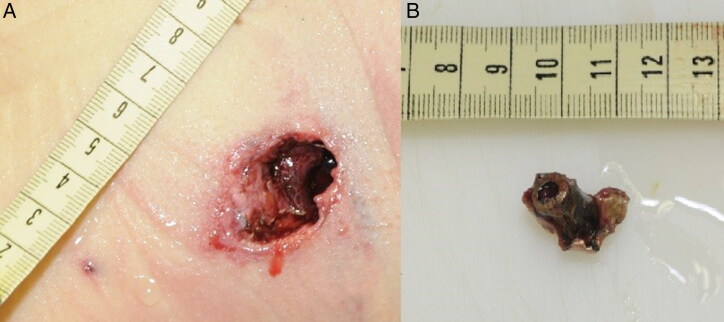
Photograph showing a projectile that had impacted intermediate obstacles before skin entrance (B), and the resulting irregularly-shaped skin wound (A).

**Figure 2.**
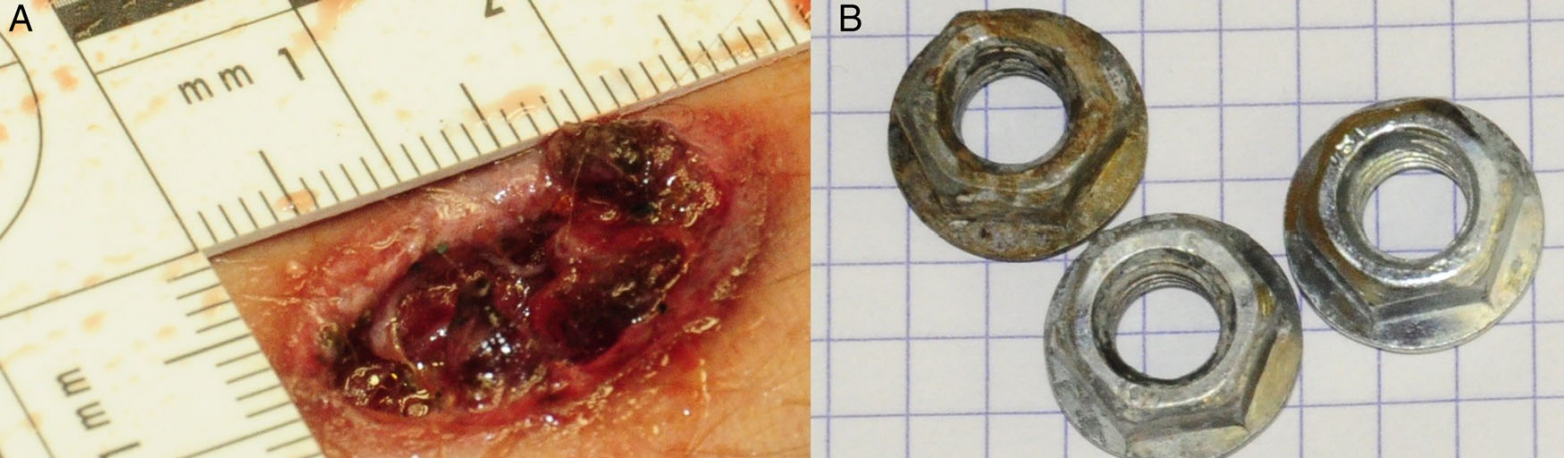
Photograph showing an irregularly-shaped skin wound (A) caused by shrapnel-like projectiles (nuts) (B) found near the shallow orifice.

**Figure 3.**
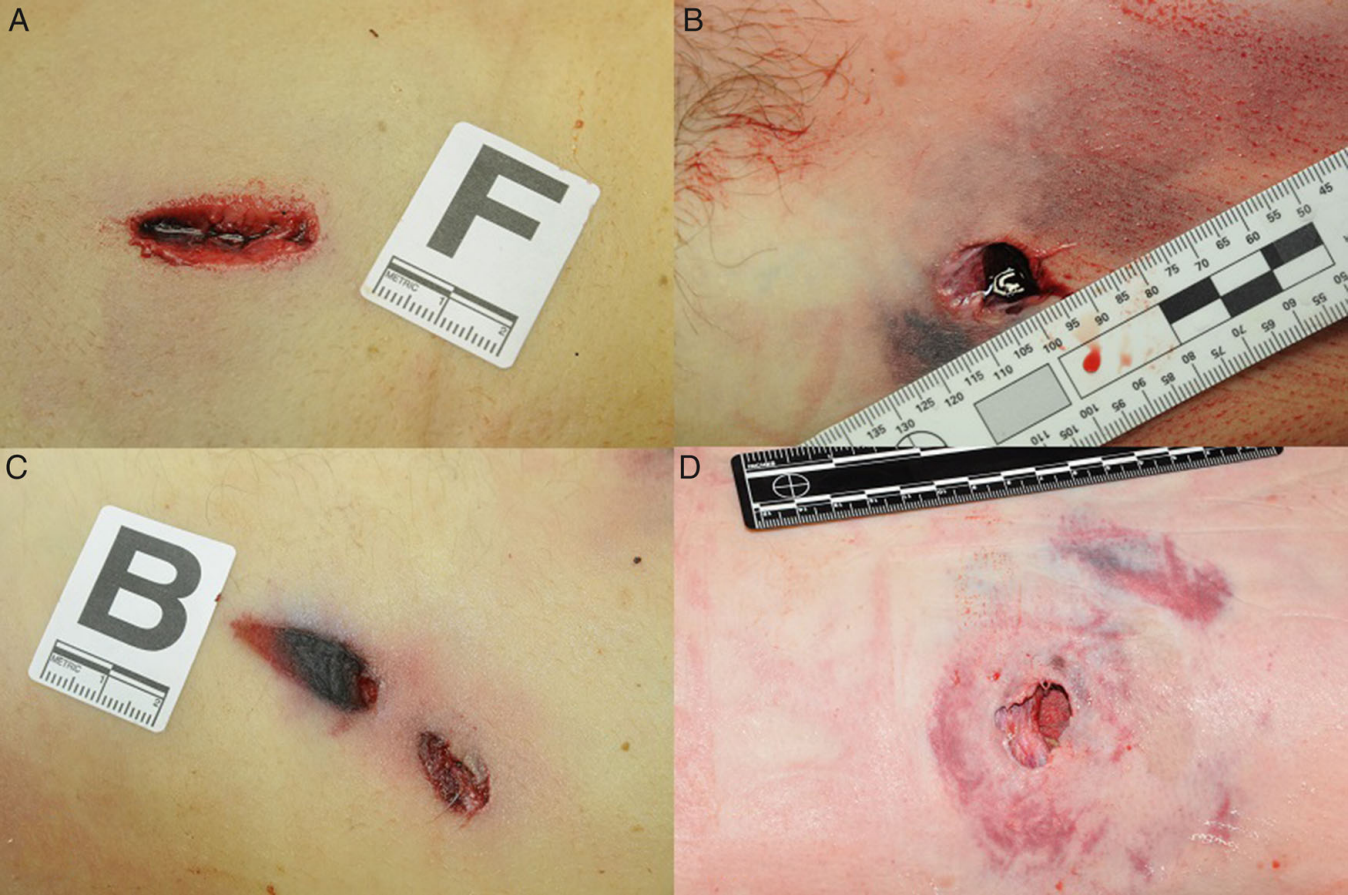
Photographs of representative ballistic entrance wounds. The projectile penetrated the skin laterally (A and C). The projectile penetrated the skin without deformation (B) and a deformed projectile penetrated the skin, causing an irregularly-shaped wound (D).

**Figure 4.**
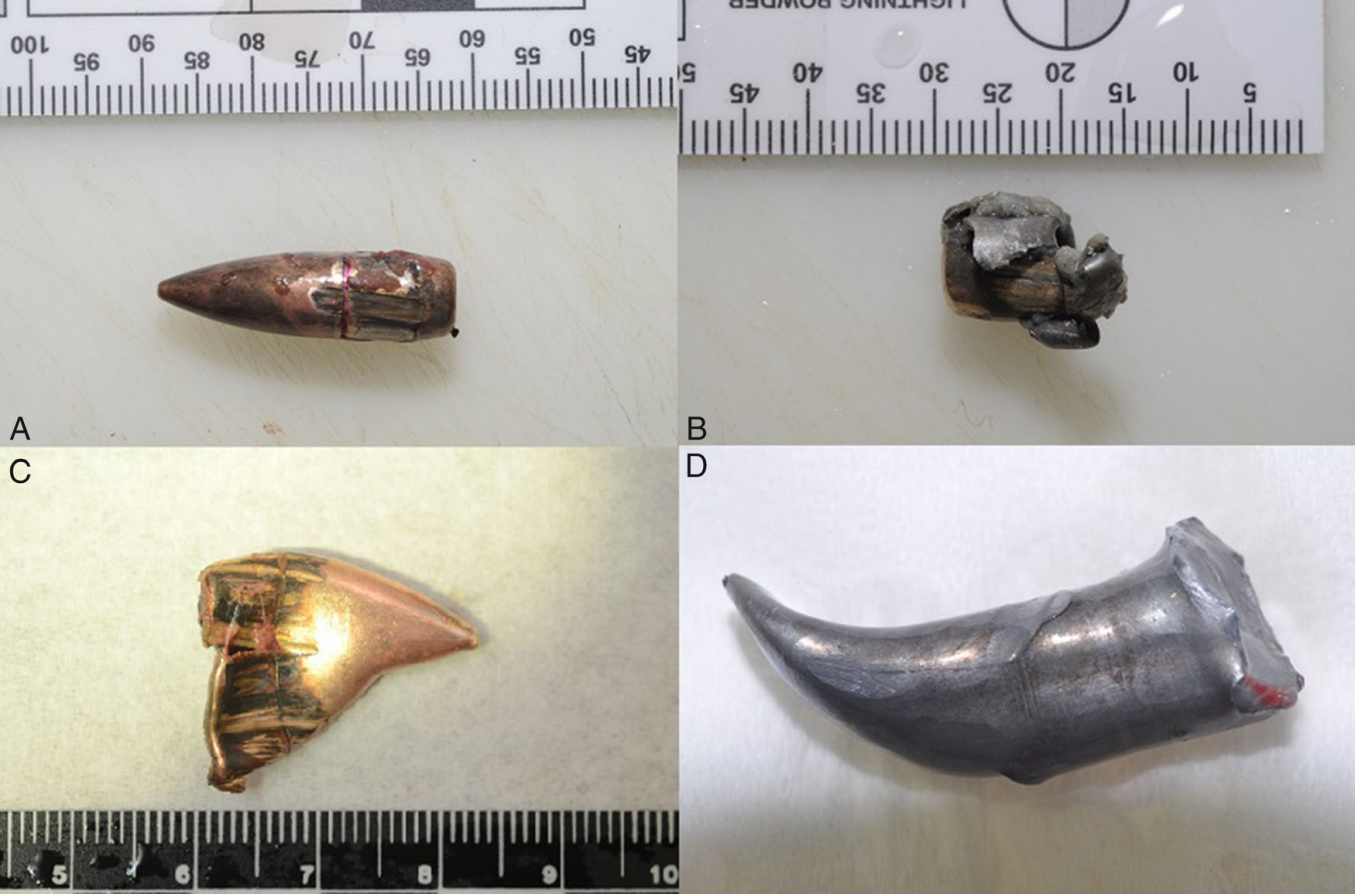
Photograph showing deformed projectiles that encountered multiple intermediate obstacles before penetrating the body. Full metal-jacketed bullet (A). Flattened bullet (B). Deformed jacket without core (C) and core without jacket (D).

Injuries caused by secondary blasts, i.e. from shrapnel-like projectiles (the bombs were loaded with nails, screws, nuts and bolts), caused many cutaneous wound patterns, which can be macroscopically very similar to those caused by firearm projectiles (Figures 1 and 2). Firearm injuries cause various cutaneous wounds (Figure 3). During the terrorist events, shots were fired with high-velocity weapons (and various projectiles) in uncontrolled directions, and projectiles encountered multiple intermediate obstacles (including the bodies of other victims), causing ricochets. Consequently, the victims were often affected by deformed projectiles (Figure 4), and lesional patterns were highly variable. Some ballistic entrance wounds were immediately suggestive of projectiles encountering intermediate obstacles or ricochets (Figure 3A and 3C: the projectiles penetrated the skin laterally), other projectiles appeared intact (Figure 3B) or were deformed and caused irregular-shaped lesions (Figure 3D).

**Figure 5.**
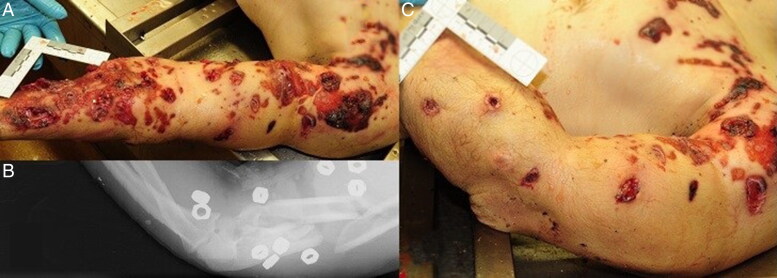
Photograph showing numerous skin wounds caused by blunt impacts with disruption and penetrating soft tissue wounds on a victim’s upper limbs (A and C), with multi-fragmentation associated fractures visible in the X-ray image (B).

**Figure 6.**
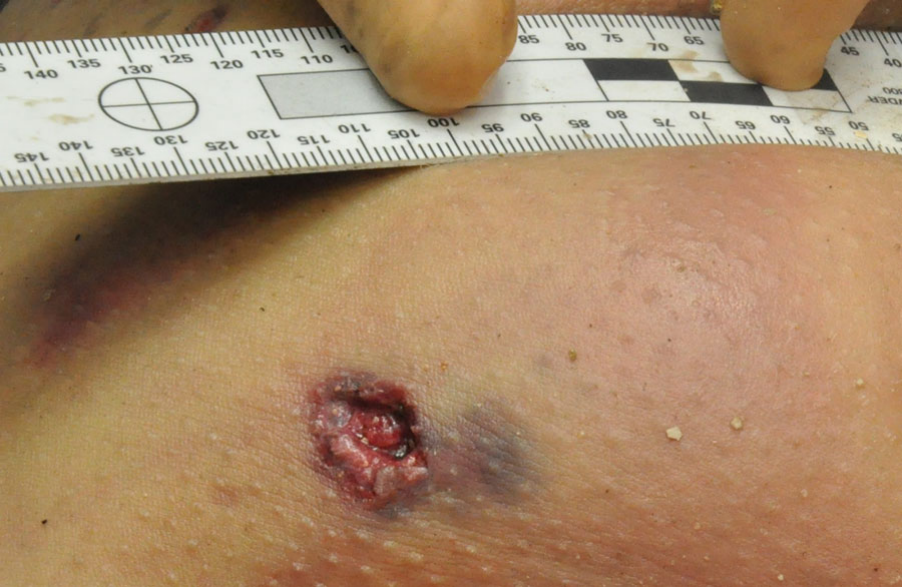
shrapnel-like injury isolated on the body.

Secondary blast injuries (nuts in our cases) caused skin injuries with irregular shapes. These skin wounds were caused by blunt impacts, with disruption and penetrating trauma to the soft tissue as well as to bone, with multi-fragmentation-associated fractures. As seen in Figure 5, shrapnel-like projectiles often caused numerous injuries, with obvious bodily distributions. These findings could make the diagnosis; however, sometimes these wounds were isolated on the body, or on body fragments (Figure 6), which made it difficult to identify the lesion by external examination of the body alone, and therefore, required performing an autopsy, imaging or even pathology.

## Discussion

When a bomb explodes, a shockwave is caused by gas expansion. As described by the Friedlander equation, this shockwave initially causes an increase in ambient pressure (the blast wave), up to a maximum, and then as the wave travels, ambient pressure decreases to negative values [5]. At the same time, overheated air moves rapidly (the blast wind). Depending on their distance from the bomb (and its carrier, if any), victims may be injured by these pressure changes, by shrapnel-like projectiles propelled through air movement or by the collapse of surrounding architectural structures, depending on the power of the explosion. Therefore, related lesions can be classified into four groups (Table 1) [6–8]. The closer victims are to the site of the explosion, the more they suffer from the blast wave. The further victims are from the blast, the more they suffer from the blast wind. Blast phenomena occur either separately or in combination, and depend on the topography of the surrounding environment (interior/exterior) and even the particular configurations of these environmental typologies (i.e. inside with low confinement or outside with nearby buildings that reflect the blast wave) [9]. Therefore, explosions can project both pieces of metal from the container of the explosive charge and added metallic material, such as ball bearings, nuts, bolts and nails. In the latter case, as highlighted here, injuries caused by firearms and those caused by exploded projectiles (nuts in our cases) can present very similar lesional patterns, as previously discussed [10]. How can these lesions be differentiated?

**Table 1.** Classification and types of blast injuries [6–8].

Classification of blast injuries	Types of blast injuries
Primary blast injury	Injuries caused by blast wave through human tissues of varying densities
Secondary blast injury	Injuries caused by blast wind that turns various objects into penetrating or blunt body projectiles
Tertiary blast injury	Projection of the whole body by the blast wind against its environment or crushing (collapse) of the environment on the body
Quaternary blast injury	Other non-specific injuries

As noted in previous studies [11, 12], during postmortem examination, projectile trauma from gunshot wounds can often be macroscopically distinguished from shrapnel-like trauma according to differences in the size, shape, number and distribution of wounds on the body. Shrapnel-like projectiles are often numerous and much more variable regarding shape and size than gunshot projectiles (Figure 5). Moreover, shrapnel-like projectiles are projected with a lower velocity (the high initial velocity is thought to be lost quickly). This results in wounds that are wider, more irregular and shallower (few shrapnel-like projectiles exit the body). In our cases, the velocity of the ballistic projectiles was great, but their mass was low; in contrast, the shrapnel-like projectiles had a low velocity but a large mass. The kinetic energy the projectiles carried could have been quite similar, with all of their impact energy released on the bodies. This may partly explain the similarity in appearance of the entrance wounds with the different projectiles.

Projectiles and tissues have a reciprocal influence in wounds. Each part of the body (e.g. skin, muscle, fat and bone) has a different density. When a projectile penetrates the body, its behaviour depends on its velocity as well as on the cross-sectional density [13]. As explained by DiMaio, kinetic energy transfer is determined by four main factors [14, 15]:

1. The amount of kinetic energy contained in a bullet;
2. The bullet’s angle of yaw, which depends on the bullet’s physical characteristics, the twist imparted by the barrel and the air density;
3. The bullet’s configuration;
4. The density, strength and elasticity of the tissue penetrated by the bullet and the length of the wound track.

During autopsy, we made lesional distinctions with these parameters in mind. The second factor explains why unstable projectiles in flight caused larger entrance wounds on impact with the body. Moreover, entrance wounds caused by ricocheting bullets tended to be larger and more irregular in shape, and the edges of the entrance hole were usually ragged and irregular [14]. On external examination, ricocheting bullets also changed the classical appearance of the entrance wounds (Figure 1). The wounds produced secondary to ricochets were penetrating rather than perforating, which can only be demonstrated by an autopsy. The four criteria (i.e. size, shape, number and distribution of the wounds) usually found in the literature for distinguishing between lesions caused by these two types of projectiles (firearm and shrapnel-like) may be difficult to use with only an external examination of corpses. This confusion is especially noticeable in confined settings because the reflection of pressure waves exposes victims to more intense and lasting pressure changes, causing additional injury [16, 17].

One of the limitations of our work was the pressure on the forensic teams.

**Figure 7.**
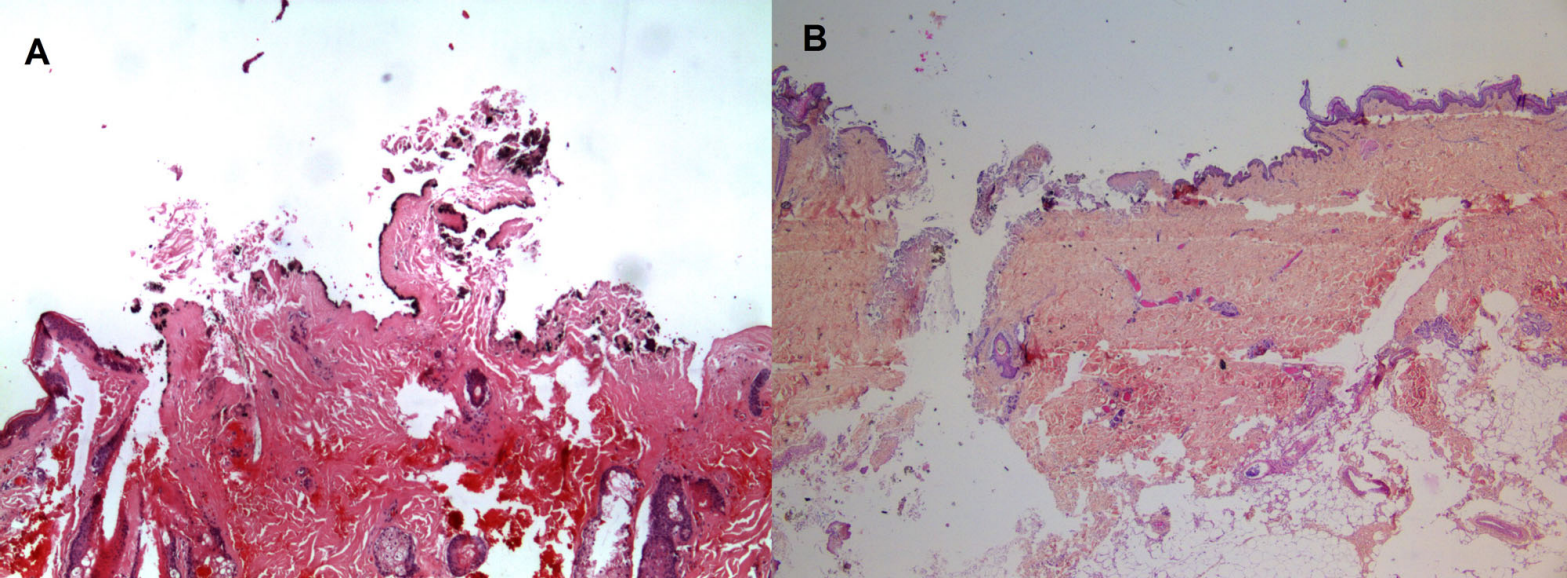
Microscopical aspects of these shrapnel-like injuries. The origin of the wounds is distinguished by the presence (A) or absence (B) of gun powder particles (hematein stain combined with an aluminum mordant, ×100).

The judicial authorities asked us to prioritise victim identification rather than determining the causes of death. A time limit was also set by the judicial and political authorities (7 days for 130 bodies). As a result, not all victims were autopsied, and some underwent only an external examination. This decision was not ours, in these very sensitive contexts (e.g. psychological, media and political). It is important in these difficult situations to benefit from systematic postmortem imaging. Standard radiography is more suitable than CT scans, which are subject to artefacts caused by metal projectiles. If possible, autopsy and then anatomopathological analyses of the orifices also help determine the cause of the wounds according to the presence of burns or gunpowder or oil particles (Figure 7).

## Conclusion

When occurring simultaneously in terrorist attacks, injuries caused by secondary blasts appear as cutaneous wound patterns that can be macroscopically very similar to those caused by firearm projectiles. The criteria usually found in the literature for distinguishing these two types of projectiles may be difficult to use.

Shrapnel-like projectiles often cause numerous injuries, with obvious bodily distributions, which could make the diagnosis. However, sometimes these wounds are isolated on the body, or on body fragments, making it difficult to identify the lesion by external examination alone; therefore, the addition of autopsy, imaging or even pathology is required. Forensic pathologists must be trained to recognise these specific wound patterns.
